# Salivary cytokine levels in early gingival inflammation

**DOI:** 10.1080/20002297.2017.1364101

**Published:** 2017-08-11

**Authors:** Daniel Belstrøm, Christian Damgaard, Eija Könönen, Mervi Gürsoy, Palle Holmstrup, Ulvi Kahraman Gürsoy

**Affiliations:** ^a^ Department of Odontology, Section for Periodontology and Oral Microbiology, Faculty of Health and Medical Sciences, University of Copenhagen, Copenhagen, Denmark; ^b^ Institute for Inflammation Research, Center for Rheumatology and Spine Diseases, Rigshospitalet, Copenhagen University Hospital, Copenhagen, Denmark; ^c^ Department of Periodontology, Institute of Dentistry, University of Turku, Turku, Finland

**Keywords:** Gingivitis, human, vascular endothelial growth factor, interleukin-8, monocyte chemoattractant protein-1, interleukin-1β, interleukin-1 receptor antagonist

## Abstract

Salivary protein levels have been studied in periodontitis. However, there is lack of information on salivary cytokine levels in early gingival inflammation. The aim of this study was to determine salivary levels of vascular endothelial growth factor (VEGF), interleukin (IL)-8, monocyte chemoattractant protein (MCP)-1, IL-1β, and IL-1 receptor antagonist (IL-1Ra) in gingival inflammation. Twenty-eight systemically and orally healthy nonsmokers abstained from oral hygiene protocols for 10 days. After that, self-performed cleaning was resumed for 14 days. Plaque and gingival indexes were measured, and saliva samples were collected at days 1, 4, 7, 10, and 24. Salivary cytokines were detected with Luminex®-xMAP™. Salivary IL-1β, IL-1Ra, and VEGF levels decreased after 10 days’ development of experimental gingivitis and reached baseline levels at the end of the 2-week resolution period. Salivary IL-8 levels decreased and remained low during development and resolution of experimental gingivitis. Initial inflammation in gingival tissues is associated with a decrease in inflammatory cytokines in saliva. Further studies are needed to evaluate if inflammatory cytokines bind to their functional receptors within the gingival tissue during early gingivitis, which may limits their spillover to the gingival crevice and ultimately saliva.

## Introduction

Gingivitis is the inflammatory gingival response against excessive bacterial biofilms [1]. It is a reversible condition. However, if left undisturbed, the gingival inflammation becomes chronic and may ultimately lead to periodontitis, with degradation of the tooth-supporting tissues.

Early detection of inflammatory changes in the gingival tissues has major significance for the treatment and prognosis of periodontal diseases. While gingivitis is easily reversed in healthy individuals, the risk of disease progression to periodontitis in susceptible individuals is substantial. At present, the diagnosis of gingivitis relies on clinical signs of gingival inflammation (i.e. gingival edema, bleeding on probing, and increased crevicular fluid) the latter of which spill over to the saliva. Based on its content of various biological substances, saliva may possess the potential to serve as a diagnostic tool for oral diseases [2]. Several salivary proteins, such as interleukin (IL)-1β, matrix metalloproteinase (MMP)-8, and pyridinoline cross-linked carboxyterminal telopeptide of type I collagen (ICTP), have successfully been used in screening for periodontal disease activity [3,4]. However, as these proteins are associated with extensive collagen degradation and osteoclast activation [5,6], they do not reflect the initiation of gingivitis.

At the molecular level, initiation and resolution of gingival inflammation are regulated by host chemokines, cytokines, and to some extent pro-resolving lipid mediators [7]. The initial steps in this inflammatory process are increased blood flow and vascularization (angiogenesis), increased vascular permeability, and migration of neutrophils and monocytes/macrophages to the site of infection [6]. The hypothesis for this study was that early-stage gingivitis impacts salivary levels of specific chemokines and cytokines. Thus, the aim was to quantify selected chemokines and cytokines associated with early stages of gingivitis (i.e. vascular endothelial growth factor [VEGF], IL-8, monocyte chemoattractant protein [MCP-1], IL-1β, and IL-1 receptor antagonist [IL-1Ra]).

## Materials and methods

### Study population and its selection criteria

A total of 31 dental students from the University of Copenhagen were enrolled. The recruitment protocol was based on the following selection criteria: systemically and orally healthy nonsmokers aged ≥18 years who were willing to discontinue brushing their teeth and interdental cleaning for 10 days. All subjects with active caries lesions, gingivitis or periodontitis, hyposalivation, any prescribed medication within the previous 3 months, including antibiotics and/or professional dental cleaning, were excluded. The volunteers were informed about the objectives of this experiment prior to obtaining their written consent. The study was approved by the regional ethical committee of the Capital Region of Denmark (H-16016368–56386) and complied with the Declaration of Helsinki. Furthermore, it was reported to the Danish Data Authority (SUND-2016–58) and registered at clinicaltrials.gov (NCT02913235).

### Study design and clinical recordings

Oral health status was screened full-mouth and by use of bitewings radiographs. The total duration of this study was 24 days, out of which participants stopped all oral hygiene protocols for 10 days. Regular oral hygiene was then resumed for 14 days. Gingival examinations, including registrations of plaque and gingival inflammation, were performed at baseline and at days 4, 7, 10, and 24 by the same examiner (DB). Plaque index (PI) and gingival index (GI) were recorded and calculated based on four sites (buccal, lingual, mesial, and distal) of six preselected teeth (16, 12, 24, 44, 32, and 36) as previously described [8,9].

### Saliva sampling

Stimulated saliva samples were collected, as previously described [10]. At each visit, the samples were collected from the study population by the same examiner (DB) between 8:00am and 6:00pm. Great care was taken that all saliva sampling per subject was performed at the same time of the day. In brief, participants were instructed to avoid eating and drinking for at least 2 h before sampling. Chewing a sterile paraffin gum was used to stimulate saliva secretion, and participants spat into a sterile plastic cup until 1 mL of sample material was collected. Saliva samples were then divided into two aliquots and immediately placed on dry ice. Samples were stored at −80°C until further analysis.

### Analysis of salivary biomarkers

After thawing, all saliva samples were centrifuged at 9300 g for 5 min. In salivary assays, the supernatant was used. Salivary concentrations of VEGF, IL-8, MCP-1, IL-1β, and IL-1Ra were detected by the Luminex® xMAP™ technique (Luminex Corporation, Austin, TX) with the commercially optimized Bio-Plex kits (pro-human cytokine group I assays; Bio-Rad, Santa Rosa, CA) according to the manufacturer’s instructions. The detection limit of the assay was 3.1 pg/mL for VEGF, 1.0 pg/mL for IL-8, 1.1 pg/mL for MCP-1, 0.6 pg/mL for IL-1β, and 5.5 pg/mL for IL-1Ra.

### Statistical analyses

All data were checked for normal distribution. Data following a Gaussian distribution were compared using repeated measures of analysis of variance and repeated *t*-test, whereas nonparametric data were analyzed with Friedman’s test with Dunn’s multiple comparison tests. *p*-Values <0.05 were considered statistically significant. Correlations between cytokines were computed using Spearman’s signed rank test and corrected for multiple dependent comparisons. GraphPad Prism v5 (GraphPad Software, Inc., La Jolla, CA) was used as statistical software.

## Results

Out of 31 recruited subjects, 29 (95.3%) completed the study. The mean age of the participants was 24.7 years (range 22–29 years), and the majority (82.8%) were female. The reason for the two dropouts was the use of antibiotics prescribed for bacterial infection unrelated to the oral cavity. Furthermore, as one saliva sample tube broke during centrifugation, the remaining samples from that participant were discarded. Consequently, samples from 28 participants were included in further analyses.

### Clinical data

**Figure 1.**
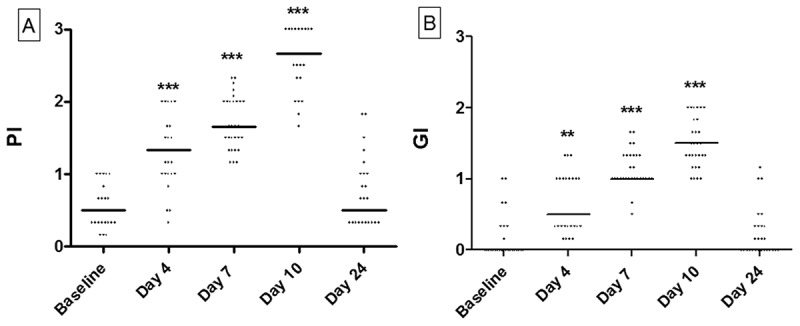
Clinical data. (a) Dot-plot of plaque index (PI) recorded at baseline and at days 4, 7, 10, and 24. (b) Dot-plot of gingival index (GI) recorded at baseline and at days 4, 7, 10, and 24. Horizontal lines: median value. **p < 0.01; ***p < 0.0001.

PI and GI values increased significantly during the experimental gingivitis period (at days, 4, 7, and 10). After 2 weeks of resolution (day 24), PI and GI scores were not significantly different from baseline scores (Figure 1).

### Early gingivitis impacts levels of salivary cytokines

**Figure 2.**
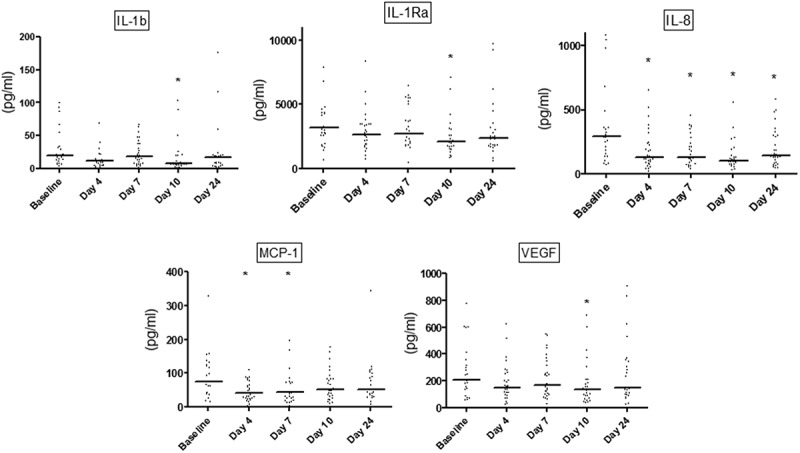
Salivary levels of interleukin (IL)-1β, IL-1Ra, IL-8, monocyte chemoattractant protein (MCP-1), and vascular endothelial growth factor (VEGF). Dot-plot of salivary levels of IL-1β, IL-1Ra, IL-8, MCP-1, and VEGF recorded at baseline and at days 4, 7, 10, and 24. Horizontal lines: median value. *p < 0.05.

In all samples, salivary concentrations of tested cytokines were above the detection limits of the test kit. Salivary levels of IL-1β, IL-1Ra, IL-8, MCP-1, and VEGF are presented in Figure 2. Salivary IL-1β, IL-1Ra, and VEGF were all significantly reduced after 10 days (*p* < 0.05) compared to baseline, whereas MCP-1 levels were reduced at day 4 and day 7, respectively (*p* < 0.05). Salivary IL-8 was significantly reduced at day 4 and stayed low, even after resolution of experimental gingivitis (*p* < 0.05; Figure 2).

### Correlation of salivary cytokines

Correlation of baseline concentrations and levels recorded at days 4, 7, 10, and 24 of each cytokine is presented in Table 1. A strong correlation was observed between baseline samples and samples collected 14 days after resolution of experimental gingivitis (day 24) of IL-1β (*R* = 0.66, *p* < 0.001), IL-1Ra (*R* = 0.85, *p* < 0.001), IL-8 (*R* = 0.79, *p* < 0.001), and VEGF (*R* = 0.80, *p* < 0.001).

**Table 1.** Correlation coefficients (*R*) for salivary IL-1β, IL-1Ra, IL-8, MCP-1, and VEGF levels between the visits at baseline and at day 4, 7, 10, and 24

	Baseline vs. day 4	Baseline vs. day 7	Baseline vs. day 10	Baseline vs. day 24
IL-1β	0.33	0.45*	0.38	0.66**
IL-1Ra	0.38	0.37	0.44*	0.85**
IL-8	0.48*	0.46*	0.53*	0.79**
MCP-1	0.63**	0.50*	0.68**	0.40*
VEGF	0.40*	0.40*	0.54*	0.80**

*p < 0.05; **p < 0.01.

IL, interleukin; IL-Ra, IL-1 receptor antagonist; MCP-1, monocyte chemoattractant protein; VEGF, vascular endothelial growth factor.

Correlation between salivary IL-1β, IL-1Ra, IL-8, MCP-1, and VEGF is detailed in Table 2. While highly significant correlations were observed between salivary levels of all cytokines investigated, the strongest correlations were observed between IL-1β and IL-8 (*R* = 0.86) and IL-1Ra and VEGF (*R* = 0.92).

**Table 2.** Correlation coefficients (*R*) between salivary levels of IL-1β, IL1-Ra, IL-8, MCP-1, and VEGF

	IL-1Ra	IL-8	MCP-1	VEGF
IL-1β	0.78***	0.86***	0.48***	0.78***
IL-1Ra		0.73***	0.58***	0.92***
IL-8			0.49***	0.76***
MCP-1				0.62***

***p < 0.0001.

No significant correlation was observed at the individual level between clinical parameters (PI and GI) and salivary levels of IL-1β, IL-1Ra, IL-8, MCP-1, and VEGF (*p* > 0.05).

## Discussion

The purpose of the present investigation was to characterize salivary levels of five selected inflammatory cytokines associated with gingivitis in order to test the hypothesis that early-stage gingival inflammation impacts salivary concentrations of specific cytokines. The main finding was that early-stage experimental gingivitis was associated with a significant decrease in salivary concentrations of all cytokines investigated.

Salivary levels of the cytokines tested were altered at different time points. MCP-1 and IL-8 were significantly decreased already at day 4, whereas VEGF, IL-1β, and IL-1Ra were significantly decreased after 10 days of oral hygiene discontinuation compared to baseline levels. These findings probably reflect the different roles of the chemokines and cytokines investigated in early inflammation. IL-8 and MCP-1 are chemoattractants, which promote the migration of neutrophils and monocytes/macrophages to the site of the infectious challenge [11–13]. Thus, the significant decrease in IL-8 and MCP-1 at day 4 is consistent with the clinical recordings, which showed moderate plaque formation but minimal signs of gingival inflammation. On the contrary, VEGF and IL-1β are cytokines that orchestrate inflammatory changes of the gingival tissues such as angiogenesis and edema [14–16]. It is therefore interesting that VEGF and IL-1β were not significantly decreased until 10 days after oral hygiene discontinuation, when the majority of the participants had developed moderate to severe gingivitis.

Other experimental gingivitis studies have demonstrated decreased or steady gingival crevicular fluid IL-8 levels [11,13], which is consistent with data from the present investigation. On the other hand, elevated IL-1β, steady IL-1Ra, and decreased MCP-1 levels have been observed in gingival crevicular fluid samples in experimental gingivitis [17–19], which conflicts with data from this report. Furthermore, in one investigation, salivary IL-1Ra levels were found to be associated with increased pocket-depth measurements in an experimental gingivitis model, while no association was observed between IL-1Ra and PI or GI [20]. These discrepancies might reflect differences between studies in terms of sample material used (saliva vs. crevicular fluid) and duration of the experimental gingivitis protocol (10 vs. 21 days). Notably, the discontinuation of oral hygiene employed in this investigation lasted only 10 days, and data suggest that in the initial phases of inflammation, preserved gingival tissue integrity supports the binding of the chemokines and cytokines investigated to limit their release into gingival crevicular fluid and saliva.

Major intra-individual differences in salivary levels of each cytokine were noted. This finding probably reflects the fact that while some individuals developed pronounced generalized gingivitis, others only showed modest clinical signs of gingival inflammation during the 10-day period of oral hygiene cessation. This is consistent with clinical data from other experimental gingivitis studies in which substantial variations in gingival inflammation were also recorded [11,21,22]. However, a good correlation between salivary levels of each cytokine recorded suggests their possible use in screening for early signs of gingival inflammation. Obviously, this is especially relevant in individuals susceptible to periodontitis.

Some potential limitations apply to the results of this study. Stimulated saliva samples were used for cytokine analysis, which means that salivary levels of the cytokines investigated were possibly diluted as a consequence of the sampling strategy [23]. However, salivary levels of each cytokine were above the detection level in all samples. Collection of unstimulated saliva samples might be influenced by tongue and cheek movements, which may impact salivary secretion and protein concentration between subjects. Consequently, stimulated saliva samples are suggested as a valid alternative to unstimulated saliva samples [24]. Partial recordings were used for calculation of PI and GI, which may have influenced correlation analysis between clinical parameters and salivary cytokine levels. Thus, future experimental gingivitis studies could employ full-mouth registration of plaque accumulation and gingival inflammation.

In conclusion, salivary levels of inflammatory chemokines and cytokines were significantly decreased during initiation and development of gingivitis. Preserved gingival tissue integrity at early phases of gingivitis may explain why pro-inflammatory cytokines are not released into gingival crevicular fluid and saliva. Further studies in susceptible groups, including smokers, diabetics, and patients with aggressive forms of periodontitis, are needed to evaluate the potential of salivary concentrations of inflammatory cytokines to be used for screening of early signs of gingival inflammation.
